# Editorial: Invertebrates as model organisms: opportunities and challenges in physiology and bioscience research

**DOI:** 10.3389/fphys.2023.1244594

**Published:** 2023-07-04

**Authors:** Natraj Krishnan, Jian Xu

**Affiliations:** ^1^ Department of Biochemistry, Molecular Biology, Entomology and Plant Pathology, Mississippi State University, Starkville, MS, United States; ^2^ Laboratory of Biology and Information Science, School of Life Sciences, East China Normal University, Shanghai, China

**Keywords:** *Drosophila melanogaster*, *Bombyx mori*, *Locusta migratoria*, neurtrophin-4, vitellogenin receptor (VgR), model system, memory and sleep, cardiac diseases

A model organism is defined as a “cognitive stand-in: instead of investigating the phenomenon directly, one studies an easier to handle alternative” (Levy and Currie, 2015). Model organisms are generally chosen for physiology and bioscience research because of some practical advantages such as ease of availability and breeding, ease of maintenance in the laboratory, short generation time and rapid development as well as their ability to respond well to experimental techniques and manipulations (Kellogg and Schaffer, 1993; Bolker, 2009). Invertebrates represent over 90% of non-plant species on the earth. While their body structure is different from vertebrate body anatomy and structure, its physiology and biochemistry offer avenues for new approaches that have only been partially explored. Strategic investments in invertebrate model organisms have resulted in major advances in our fundamental understanding of living systems, health and disease as well as have offered technological tools to further advances in bioscience research. As such, invertebrate study systems have been fundamental for most advances in biological and biomedical research, providing key insights into varied fields from genetics to behavioral ecology (Krishnan et al., 2023). While the fruit fly *Drosophila melanogaster*, the nematode *Caenorhabditis elegans* and the fungus *Saccharomyces cerevisiae*, have served as pre-eminent invertebrate models (Strange, 2007; Gilbert, 2008; Karathia et al., 2011) for the past several decades, there are newer invertebrate models (*Bombyx mori*—Meng et al., 2017; *Locusta migratoria*—Siddiqui and Khan, 2021) emerging that have led to increased understanding of developmental physiological processes in the organism itself as well as in higher animals.

This Research Topic focuses on some of the current opportunities and challenges in physiology and bioscience research utilizing invertebrates as model systems and is a compilation of three original research articles, one perspective article and one review article.

Sevoflurane and Isoflurane are the most commonly used general anesthetics in the course of surgery, yet the mechanisms responsible for their effects, or the neuronal changes that underlie them or the signaling processes that potentiate faster recovery from sevoflurane anesthesia compared to isoflurane are largely unclear. Ma et al., demonstrate that the Janus kinase (JAK) pathway mediates faster recovery from sevoflurane anesthesia compared to isoflurane anesthesia. The authors used a combination of techniques including RNA-seq, RNAi and qRT-PCR followed by Western blotting to demonstrate this process using migratory locusts (*L. migratoria*) as a model system to unveil the underlying molecular mechanism. This demonstrates that migratory locusts can be effectively used as animal models to study the mechanisms underlying anesthesia and post-anesthesia recovery in higher animals including humans.

The domestic silkworm *B. mori* has a long history with humans, spanning over 5,000 years. It is primarily known for silk production and has played a crucial role in the economic growth of many countries. In the nineteenth century, it became a model organism for scientific research, providing important insights in genetics and molecular biology. While it remains significant in sericulture worldwide, its importance has further increased due to genome sequencing, annotation, and the development of genetic technologies (Xia et al., 2014). *B. mori* larvae can serve as living bioreactors, capable of producing valuable proteins, therapeutics, and silk-based biomaterials when genetically modified. Zhang et al., report on the generation of transgenic silkworms bioengineered to produce human neurotrophin-4 (NT-4). NT-4 belongs to the neurotrophin family of growth factors, and has shown potential for playing an important role in repairing nerve injury. There is an increasing demand for NT-4 in biomedical applications and this work demonstrates the development of a novel silk protein-based functional silk material in which human NT-4 is expressed in the silk gland of the transgenic silkworm.

In their review article, Marquand et al., delve into the physiological mechanisms that modulate acquisition and consolidation of memory and how research on the fruit fly *D. melanogaster* has paved the way to understanding regulation of memory by sleep. A judicious discussion of the recent advances in the field and how such findings also point to its potential therapeutic applications has been provided. Additionally, the authors also highlight some important unanswered questions that would direct future research on the interaction between memory and sleep.

Xu et al., through a series of elegant experiments demonstrate that the vitellogenin receptor in *B. mori* transports the 30 kDa protein LP1 without the cell-penetrating peptide into the oocytes thereby promoting egg growth and embryonic development. Vitellogenin receptors (VgR) play a decisive role in vitellogenesis and uptake of vitellogenin by oocytes during its development. During vitellogenesis, production of yolk resources is essential for egg maturation and helps in embryo development after egg laying. This work demonstrates that in addition to transporting Vg, the VgR in *B. mori* also transports BmLP1. However, the amino acid residue responsible for binding BmLP1 to BmVgR remains unidentified. This work paves the way to a greater understanding of many processes that utilize similar transport mechanisms and the range of application spans from pest control to targeted drug delivery in biomedicine.

The fruit fly *D. melanogaster* has become a valuable model for studying cardiac diseases, including developmental abnormalities and functional impairment in adults. By utilizing classical and molecular genetics, researchers have made significant progress in understanding the development of the fly heart. This research has been crucial in identifying key signaling events involved in cardiac field formation, cardiomyocyte specification, and the formation of a functional heart tube. Zhao et al., focus on the functional and physiological similarities between fly and human hearts in their perspective article. They also highlight future prospects of leveraging the physiology of *Drosophila* as a model system for studying human cardiac diseases.

To summarize, the Research Topic comprises a Research Topic of articles contributed by various experts in the field of invertebrate physiology. These articles not only explore the applications of invertebrate species in physiology and bioscience research but also delve into specific methodologies. By doing so, they not only expand our existing knowledge but also offer fresh insights and perspectives on the opportunities and challenges that lie within the realm of physiology and bioscience research. These articles serve as valuable resources for researchers seeking to advance their understanding of invertebrate physiology and explore new avenues within the field.
